# Iatrogenic femoral nerve injuries: Analysis of medico-legal issues through a scoping review approach

**DOI:** 10.1016/j.amsu.2021.103055

**Published:** 2021-11-10

**Authors:** Filippo Gibelli, Giovanna Ricci, Ascanio Sirignano, Paolo Bailo, Domenico De Leo

**Affiliations:** a Department of Diagnostics and Public Health, Section of Forensic Medicine, University of Verona, Verona, Italy; b Section of Legal Medicine, School of Law, University of Camerino, Camerino, Italy

**Keywords:** Femoral nerve palsy, Iatrogenic injury, Complications, Neuropathy

## Abstract

**Purpose:**

Accidental femoral nerve injury is a well-known iatrogenic complication of orthopaedic, abdominal, and pelvic surgery. Because of the largely transitory nature of the symptoms associated with nerve damage, its true incidence is in all likelihood underestimated. This work aims to illustrate the surgical contexts within which this nerve injury is reported, based on the evidence obtained from a Scoping Review of the literature of the last 20 years, with specific reference to the underlying etiopathogenetic mechanisms and prognostic outcomes, to highlight the evaluation issues of medico-legal interest related to this pathology.

**Methods:**

We conducted a Scoping Review of iatrogenic femoral nerve injuries reported between 2000 and 2021 by searching the electronic databases Pubmed, Scopus, Ovid Medline, Ovid Emcare, and Web of Science.

We conducted the review according to the five-step methodology outlined by Arksey and O'Malley.

**Results:**

The literature search identified 104 papers, including case reports, case series, and retrospective studies. Surgical contexts within which iatrogenic femoral nerve injuries were reported include orthopaedic, abdominal, gynaecological, urological, vascular, and plastic surgery, as well as locoregional anaesthesiological procedures. The long-term prognosis was generally favourable.

**Conclusions:**

Because of its frequent iatrogenic genesis, femoral nerve injury is a topic of intense medico-legal interest. From the perspective of estimating the patient's disability, the mostly favourable nature of the prognosis makes the medico-legal assessment, in some respects, complex, thus requiring a precise evaluation methodology.

## Highlights

• Femoral nerve injuries have an iatrogenic aetiology in most cases.
• The surgical contexts in which the damage most frequently occurs are orthopaedic, abdominal and gynaecological surgery.
• The prognosis of nerve damage is in the vast majority of cases good, as it is almost always neuroapraxic.
• TThe favourable prognosis of the injury implies that its medico-legal assessment requires a very rigorous methodology.

## 1 Background

Iatrogenic femoral nerve damage is a relatively rare complication of orthopaedic, abdominal, and pelvic surgery.

However, femoral nerve injury has an iatrogenic aetiology in as many as 60 % of cases, according to data obtained from large-scale studies [1].

The first case of postoperative femoral palsy was described in 1896 by Gumpertz, who reported a case of post-hysterectomy femoral neuropathy [2].

The six surgical contexts in which iatrogenic lesions of the femoral nerve are most frequently identified are as follows: orthopaedic surgery (mainly hip replacement surgery), abdominal surgery (mainly colorectal surgery, renal transplantation and herniorrhaphy surgery), gynaecological surgery (mainly hysterectomy), urological surgery, endovascular procedures (mainly femoral catheterisation), and anaesthesiological procedures (peripheral nerve blocks).

The following main mechanisms of nerve damage are identified: purely mechanical damage from stretching, traction, compression, or dislocation (in most cases, this is simple neuroapraxic damage); damage from complete nerve transection (neurotmesic damage with a poor prognosis); damage from accidental suturing or entrapment in staples; ischaemic damage (mostly secondary to compression); heat damage (such as in the case of cement extrusion from hip prostheses, where there is an exothermic reaction with heat release during cement polymerisation); and toxic damage.

It is a condition characterised by a widely variable spectrum of severity, with the degree of functional impairment varying depending on the location and the extent of the anatomical damage, as well as the pathogenetic mechanism.

Nerve damage is mostly neuroapraxic or axonotmesic. Neurotmesic damage occurs infrequently.

In the case of neuroapraxic and axonotmesic lesions, functional damage tends to be partial and mostly self-limiting. In contrast, in the case of neurotmesis following a complete nerve section, the anatomical-functional impairment accounts for a morbid state with a dramatic impact on the patient's quality of life.

In general terms, the long-term prognosis is good, with scientific evidence of motor and sensory recovery usually occurring within 6–12 months of rehabilitation treatment.

The latest Systematic Review on iatrogenic femoral nerve injury dates back to 2010 and includes 38 scientific papers published between 2000 and 2010 [3].

Given the significant medico-legal implications of the condition, we felt it would be useful to conduct an updated literature review.

In particular, the present work aims to illustrate fully and in an updated way the characteristics of iatrogenic injuries of the femoral nerve by investigating the scientific literature on the subject of the last 20 years.

The surgical context, probable pathogenesis, clinical presentation, prognosis, and the strategies through which the damage could have been avoided were studied for each case found in the literature.

Finally, the collected data were processed to outline the most significant medical-legal implications of nerve injury, which, especially because of the overall favourable prognosis, is of significant interest in terms of healthcare litigation.

### 1.1 Anatomy and physiology of the femoral nerve

The femoral nerve is the largest branch of the lumbar plexus, originating with three roots from it.

The upper root originates from the anastomotic loop between L2 and L3, the middle root from L3, and the lower root from L4.

It is a mixed sensory and motor nerve responsible for the motor innervation of the anterior muscles of the thigh and the sensory innervation of the skin of the anterior aspect of the thigh and the anteromedial part of the leg, as well as the acetabulofemoral joint and the knee joint.

The nerve trunk emerges at the level of the fifth lumbar vertebra, along the lateral margin of the psoas major muscle, and heads caudally until it crosses the inguinal ligament, which it transits dorsally, immediately lateral to the iliopectineal arch, running along the neuromuscular lacuna together with the iliopsoas muscle [4].

In the abdominal pathway, the nerve provides some collateral branches: the nerve to the psoas major, the nerve to the iliacus muscle, the nerve to the pectineus muscle, and the nerve to the femoral artery, which originates proximal to the inguinal ligament and accompanies the vessel to the mid-thigh.

Shortly after passing the inguinal ligament, it bifurcates into two divisions, one anterior and one posterior, separated by the lateral circumflex femoral artery [5].

The branches of the anterior division are the lateral femoral cutaneous nerve and the medial femoral cutaneous nerve.

Those of the posterior division are the four motor nerve branches destined to the same number of parts of the quadriceps femoris muscle, and a purely sensory nerve, the saphenous nerve, which, at the level of the medial epicondyle of the femur, divides into its two terminal branches, the tibial and the infrapatellar branch [6].

Concerning vascularisation, the nerve has triple blood support: at the pelvic level, it is vascularised by the iliolumbar artery, at the inguinal level by the deep circumflex iliac artery, and in the thigh by the lateral circumflex femoral artery.

It is important to note that the middle femoral nerve, within the pelvic cavity, has a relatively poor blood supply, being a “watershed area” supplied by both the iliolumbar artery (a branch of the internal iliac artery) and the deep circumflex artery (a branch of the external iliac artery) [3].

In a 1987 study of 10 cadavers, Boontje and Haaxma found a disproportion in blood supply between the right and left sides [7].

In particular, the researchers observed that the left femoral nerve was more prone to an ischaemic insult because of the greater vascular supply from the right deep circumflex iliac artery than the contralateral one; the right artery, moreover, was found to have a more significant number of anastomotic connections with the iliolumbar and fourth lumbar arteries.

Finally, in the case of anatomical variants, the only one reported in the literature is the nerve entering the thigh between the artery and the femoral vein [8].

### 1.2 Femoral nerve injury: clinical and diagnostic aspects

Clinically, femoral nerve injury is characterised by both motor and sensory deficits.

As far as motor function is concerned, paresis or paralysis of the quadriceps muscle as a result of nerve damage leads to severe limitation or abolition of knee extension. The patient, who generally has a conspicuous hypotrophy of the quadriceps, has great difficulty climbing stairs or facing a slight slope, and although he can walk with his knee extended, he falls at the slightest flexion of it.

A practical and widely used system for the most objective definition of the residual motor function of the quadriceps muscle after femoral nerve injury is the MRC (Medical Research Council) muscle grading system [9], according to which 0 corresponds to no contraction and 5 indicates normal muscle strength.

However, an even more specific muscular strength assessment system is explicitly designed for estimating the residual strength of the quadriceps after femoral nerve injury. The LSUHSC (Louisiana State University Health Sciences Centre) muscle grading system provides a grading from 0 (motor activity of the muscle completely abolished) to 6 (fully preserved motor activity) [10].

Another typical motor impairment associated with nerve injury is weakness in hip flexion, which is attributable to paralysis of the iliacus and sartorius muscles.

With regard to sensibility, femoral nerve lesions are associated with sensory disturbances (hypo/anaesthesia or paraesthesia) involving the nerve's distribution territory, in other words, the front of the thigh and the inside of the knee, leg, and foot.

The patellar reflex is markedly reduced or completely abolished.

The diagnostic workup includes nerve conduction studies (NCS) and needle electromyography (EMG), as well as imaging investigations (CT and MRI) to identify and locate any compression and to define the aetiology of the disorder.

## 2 Methods

The approach that responds most effectively to the needs of a Scoping Review is that proposed by Arksey and O'Malley [11], who argued that scoping reviews can be implemented to examine the extent, scope, and nature of the literature to identify areas of research where evidence is scarce, to determine the need for a subsequent Systematic Review, to summarize and disseminate current knowledge, or to uncover gaps and direct future research.

This methodological approach requires the study to be carried out in five phases:

Step 1: identifying the research questions
Step 2: identifying relevant studies
Step 3: selecting studies
Step 4: charting data
Step 5: collating, summarizing, and reporting results

In the present work, we used the methodological approach of the Scoping Review to provide a comprehensive overview about current knowledge of iatrogenic femoral nerve injuries.

We conducted this research not only to outline the state of the art on the topic as it emerged from the study of the scientific literature, but also to identify possible injury prevention strategies and to outline an effective medico-legal assessment methodology.

The work was carried out in accordance with the operational methodology outlined in the PRISMA 2020 statement [12], which is an updated guide (replacing the previous 2009 version) aimed at indicating the best strategy to identify, select, evaluate and synthesize studies from systematic reviews and scoping reviews.

The research was registered with the Unique Identification Number “researchregistry7337”.

The registration can be accessed through the following hyperlink: https://www.researchregistry.com/browse-the-registry#home/registrationdetails/618580c8d838f80020aa3a92/.

Although the present study is a Scoping Review and not a Systematic Review, we equally verified the adequacy of the methodology conducted through the AMSTAR2 criteria (electively directed at assessing the methodological adequacy of systematic reviews) [13].

The level of compliance was low, in our opinion, due to the particular type of research conducted, namely a Scoping Review, which requires a different methodological approach than that of a Systematic Review.

### 2.1 Identifying the research questions

We developed the research questions in accordance with the population/concept/context (PCC) framework, proposed by the Joanna Briggs Institute [14].

We based the formulation of the review questions on the methodology outlined by the JBI, as the application of the PCC framework is universally recognized for its ability to optimally respond to the characteristic need of a Scoping Review to address the issue through a more general approach than that of a Systematic Review [15].

We formulated four main, wide-ranging questions:

1. What are the main etiopathogenetic mechanisms of femoral nerve damage?
2. What is the overall prognosis of such injuries?
3. What are the possible preventive strategies?
4. What are the medico-legal implications for the assessment of permanent disability?

### 2.2 Identifying relevant studies

#### 2.2.1 Databases

We searched five electronic databases: Pubmed, Scopus, Ovid Medline, Ovid Emcare, and Web of Science.

We voluntarily omitted to include the databases for the grey literature search in order to avoid contaminating the research with papers with uncertified scientific validity.

#### 2.2.2 Inclusion criteria: the application of the PCC framework

Regarding the subject, we did not use the field “population” as a search criterion, since our research aimed to investigate iatrogenic lesions of the femoral nerve in general terms, without reference to specific classes of patients.

About the field “concept”, we used the concept of anatomical injury of the femoral nerve, while with regard to the field “context”, we selected the surgical field, since we wanted to specifically investigate iatrogenic nerve injuries, i.e., damages caused during invasive medical practices.

Table 1 illustrates the application of the PCC framework to the Scoping Review question.

**Table 1:** The PCC framework (inclusion criteria).

	Main Concept	Alternate keywords
Participants	–	–
Concept	Injury	Damage, Lesion, Neuropathy
	Femoral nerve	–
Context	Surgery	Surgical, Iatrogenic, Iatropathic

#### 2.2.3 Search strategy

In accordance with the methodological approach suggested by the Joanna Briggs Institute [16], our first step consisted of a preliminary search within the Ovid Medline database.

For each PCC element, we introduced the relevant keywords, and then we joined the lines related to them to obtain an overall set line for that specific PCC element, combining them with the “OR” Boolean operator.

Finally, we combined all overall set lines with the “AND” operator, in order to find the results that addressed our PCC elements.

We limited the search to the period between January 1, 2000 and March 31, 2021.

We obtained 433 resulting articles.

Table 2 shows the search strings used to search the Ovid Medline database.

**Table 2:** Search strings used to search the Ovid Medline database.

String 1	(Femoral nerve)
String 2	(((injury) OR (lesion)) OR (neuropathy)) OR (damage)
String 3	(((iatrogenic) OR (iatropathic)) OR (surgery)) OR (surgical)
Search query	((femoral nerve) AND ((((injury) OR (lesion)) OR (neuropathy)) OR (damage))) AND ((((iatrogenic) OR (iatropathic)) OR (surgery)) OR (surgical))

We then applied the same methodological approach – made the needed adjustments to keywords – on the databases Pubmed (2020 resulting articles), Scopus (4760 resulting articles), Ovid Emcare (338 resulting articles), and Web of Science (800 resulting articles).

Overall, we found 8351 articles using the above search terms and databases.

We completed the last search on May 30, 2021.

### 2.3 Selecting studies (screening phase)

Once we completed the bibliographic collection phase, we entered the 8351 articles obtained from the five databases into EndNote software.

The first and preliminary phase consisted of utilising an automatic software tool (and consequent elimination) to identify duplicate articles (n. 2544), articles not written in English (n. 326), and veterinary medicine reports (n. 107).

At the end of the initial skimming procedure, we obtained a library of 5374 articles.

We again used EndNote software for the initial screening phase, eliminating articles that were unrelated to the purpose of the Scoping Review, thus reducing the total number of articles to 545.

Using an automated system based on automatic title and abstract analysis, we discarded 4659 articles as not useful for the Scoping Review.

Thereafter, the fourth and fifth authors independently read the abstracts of the remaining 715 articles and eliminated articles that did not meet the established inclusion criteria (n 611), leaving 104 articles for full-text review.

The following inclusion criteria were adopted at this stage:

1. Case reports, case series, or retrospective studies
2. Papers specifically addressing femoral nerve injuries
3. Articles specifically concerning iatrogenic injuries
4. Articles addressing the probable etiopathogenesis of nerve injury
5. Articles containing data regarding the clinical outcomes of the injury in at least the following three months

### 2.4 Charting data

In order to get a clear view of the research results, we used a form of data graph using the Excel programme.

We decided to extract the following data from the selected individual articles:

1. Bibliographic reference
2. Surgical procedure during which the damage occurred
3. Frequency (in case of retrospective analysis)
4. Laterality of injury (uni/bilateral)
5. Pathogenesis of injury as hypothesised by the authors
6. Clinical presentation
7. Prognosis

### 2.5 Collating, summarizing, and reporting results

We summarised the results of the 104 selected papers by drawing up a table with the items used for the data charting phase.

We organized the results into seven tables, each relating to a different surgical field: orthopaedic surgery (Table 3), abdominal surgery (Table 4), gynaecological surgery (Table 5), urologic surgery (Table 6), vascular surgery (Table 7), anaesthesiological procedures (Table 8), and plastic surgery (Table 9).

**Table 3:** Reports of iatrogenic femoral nerve injury in orthopaedic surgery (2000–2021).

Reference	Procedure	Frequency	Uni/bilateral	Probable pathogenesis	Clinical presentation	Outcome
Mihalko et al. [18]	Primary cementless total hip arthroplasty	Case report	Unilateral	Nerve stretching due to the lengthening of the operated limb	Painful neuropathy in the operated limb, with radiating pain involving the anterior surface of the thigh, the lateral surface of the knee, and the foot; spasms and burning also reported	Full recovery during the postoperative period of revision surgery (trochanteric advancement procedure)
Fokter et al. [19]	Primary cementless total hip arthroplasty	Case report	Unilateral	Nerve compression by an intrapelvic mass produced as a result of a foreign-body reaction to wear debris	Thigh and groin pain, radiating below the knee; quadriceps hyposthenia; anteromedial thigh hypoesthesia; patellar reflex abolished	Almost complete resolution at 6 months after revision surgery (prosthesis replacement)
Jerosch [20]	Primary cemented total hip arthroplasty	Case report	Unilateral	Extrusion of bone cement into the pelvis (heat-related damage due to polymerisation of cement)	Knee instability when walking; inability to actively extend the knee; numbness in the anterior surface of the leg	ND
Zwolak et al. [21]	Primary cemented total hip arthroplasty	Case report	Unilateral	Extrusion of bone cement into the pelvis (heat-related damage due to polymerisation of cement)	Persistent pain in the groin; weakness in hip flexors and adductors; decreased sensation in the medial thigh	Almost full recovery 4 weeks after revision surgery (surgical removal of the cement)
O'Brien et al. [22]	Primary cemented total hip arthroplasty	Case report	Unilateral	Extrusion of bone cement into the pelvis (compression damage)	Excruciating pain in the groin, radiating to the lateral surface of the hip; numbness in the anterior and medial surface of the thigh	6 months after revision surgery (cement removal), complete resolution of pain but persistent hyperesthesia in the medial surface of the thigh
Wilson et al. [23]	Primary cemented total hip arthroplasty	Case report	Unilateral	Extrusion of bone cement into the pelvis (compression damage)	Groin pain; numbness along mid-thigh and knee; hip held in flexion; painful limitation of hip movements; hypoesthesia at L2-L3 dermatomes	Almost complete recovery after 6 months, although minimal thigh hypoesthesia persisted
Liman et al. [24]	Primary total hip arthroplasty	Case report	Unilateral	Medial nerve dislocation secondary to non-infectious iliopectineal bursitis	Subacute paresis of limb; dull sensation in the inner thigh, knee, and lower leg; inability to walk unassisted	Almost complete resolution 3 months after revision surgery (bursectomy)
Kim et al. [10]	Primary total hip arthroplasty	Case series (10 cases)	Unilateral	Post-surgical haematoma; methylmethacrylate encapsulation and polymerisation heat damage; direct trauma from contact with surgical instruments; compression from contact with bony prominences or dislocated prostheses	Complete functional deficit in all cases	Good motor recovery in all patients (3 treated with neurolysis, 1 with end-to-end suturing, and 6 with graft repair)
Farrell et al. [25]	Primary total hip arthroplasty	0.01 % (3/27,004 joints)	Unilateral	ND	2 complete and 1 incomplete lesion	Only 1 patient described: partial recovery after external neurolysis
Macheras et al. [26]	Primary total hip arthroplasty with anterior minimally invasive surgery (AMIS) technique	0.26 % (4/1512 joints)	Unilateral	Nerve injured by the retractors used during acetabular preparation	Weakness of hip flexion and knee extension; numbness over the anteromedial aspect of the thigh; hyperaesthesia and pain	3 permanent damages, 1 transient
Siguier et al. [27]	Primary total hip arthroplasty (anterior supine intermuscular approach)	0.19 % (2/1037 joints)	Unilateral	Improper retractor placement	ND	Full resolution after 9 and 12 months respectively
Eskelinen et al. [28]	Cementless primary total arthroplasty in patients with congenital high hip dislocation	1.3 % (1/75 joints)	Unilateral	ND	ND	Incomplete recovery (80 % of quadriceps strength compared to contralateral at 11.9 years follow-up)
Hallert et al. [29]	Primary total hip arthroplasty with anterior minimally invasive surgery (AMIS) technique	0.5 % (1/200 joints)	Unilateral	Compression by retractors	ND	Full resolution after 6 months
Matta et al. [30]	Primary total hip arthroplasty (anterior approach on an orthopaedic table)	0.20 % (1/494 joints)	Unilateral	ND	ND	Transient palsy
Gogus et al. [31]	Primary total hip arthroplasty with double acetabular screw placement	Case report	Unilateral	Compression by iliacus muscle haematoma secondary to heparin anticoagulation	Numbness in the medial region of the knee; groin pain; hyposthenia of the quadriceps and hip flexors; hypoesthesia in the femoral nerve territory; absent patellar reflex	Full recovery within 3 months (haematoma treated conservatively)
Darmanis et al. [32]	Primary total hip arthroplasty (direct lateral approach)	Case report	Unilateral	Incorrect positioning of a retractor, which resulted in excessive exposure of the soft tissues, that were macerated during the insertion of the threaded acetabular cup	Reduced active knee extension at 2 months after surgery; quadriceps atrophy and complete abolition of knee extension at 6 months	ND (likely no improvement, given the complete nerve damage)
Fleischman et al. [33]	Primary total hip arthroplasty	0.21 % (36/17,350 joints)	Unilateral	Established only in 1 case (compression by a large haematoma)	Complete or almost complete paralysis in 31 patients; incomplete paralysis in 5 patients; sensory symptoms in 34 out of 36 patients	Complete recovery of motor function in most patients within 6 months to 2 years; persistence of permanent sensory disturbances in most patients (80 %)
Hoshino et al. [34]	Primary total hip arthroplasty (direct anterior approach)	1.1 % (3/273 joints)	Unilateral	In one case excessive leg-lengthening; in one case improper positioning of a retractor; in one case, unidentified cause	Total abolition of quadriceps motor function in all cases	Complete motor recovery within 1 year in all cases
Lee and Marconi [35]	Primary total hip arthroplasty (direct anterior approach)	0.09 % (11/11,810 joints)	Unilateral	ND	ND	ND
Tani et al. [36]	Cemented revision total hip arthroplasty	Case report	Unilateral	Compression of the nerve by the reinforcement ring and extruded bone-cement	Numbness at the level of the anteromedial surface of the thigh; severe pain in the groin, exacerbated by hip extension; total loss of motor function of the quadriceps; anaesthesia in the territory of the femoral nerve	Nearly complete recovery 8 months after neurolysis
Nakamura et al. [37]	Cementless revision hip arthroplasty	Case report	Unilateral	Nerve compression by haematoma of the iliac muscle secondary to a pseudoaneurysm of a branch of the superior gluteal artery, occurring on an extension of a screw fixing the reinforcement plate	Severe pain in the groin; numbness in the anteromedial surface of limb; inability to walk; hip in flexion position; quadriceps hyposthenia	Full recovery within 2 years after revision surgery (transcatheter arterial embolisation and haematoma removal)
Ha et al. [38]	Cementless revision hip arthroplasty with double acetabular screw placement	Case report	Unilateral	Nerve compression by iliacus haematoma	Numbness in the medial aspect of the knee; groin pain; hypoesthesia in femoral nerve distribution; quadriceps hyposthenia	Full recovery after surgical removal of the haematoma
Fritzsche et al. [39]	Total hip arthroplasty with single acetabular screw placement	Case report	Unilateral	Unusual positioning of the screw due to the presence of an acetabular roof cyst, which resulted in permanent mechanical irritation of the iliac muscle and subsequent erosion of a muscular vessel, with consequent formation of a haematoma	Paresis and hyposthenia of the hip flexors and quadriceps; hypoesthesia of the anterior surface of the thigh; abolition of the patellar reflex	Almost full recovery at 6 weeks after revision surgery
Harvie et al. [40]	Metal-on-metal hip resurfacing arthroplasty	Case series (2 cases)	Unilateral	Compression by a pseudotumoral mass formed in response to type IV hypersensitivity local reaction to metal wear debris	Reduced hip flexion (MRC Grade 3); reduced knee extension (MRC Grade 2); anaesthesia over the distribution of anterior cutaneous branches	Poor recovery: 1 year after excision of the pseudotumor, severe denervation of the iliopsoas and vastus lateralis was observed, with little chance of clinical improvement
					Worsening hip and knee pain; reduced hip flexion (MRC grade 2); reduced knee extension (MRC grade 1); paraesthesias in the anterior surface of the thigh	No recovery after pseudotumor excision surgery
Leung and Kudrna [41]	Metal-on-metal total hip arthroplasty	Case report	Unilateral	Compression by a pseudotumoral mass formed in response to both a type IV hypersensitivity local reaction to metal wear debris and a cytotoxic reaction	Hyposthenia of iliopsoas and quadriceps; leg weakness; numbness and paraesthesias of the anteromedial surface of thigh; hypoesthesia in the distribution of L2 and L3 dermatomes	Worsening of symptoms 10 months after revision arthroplasty (debridement of the pseudotumor and head and liner exchange). An MRI showed an increase in the size of the pseudotumor, despite the removal of the offending metal-on-metal joint. Laparoscopic excision of the pseudotumor was then performed. At 35 months postoperatively, the patient had fully restored motor function, although paraesthesias persisted in the anteromedial region of the thigh
Clarke et al. [42]	Hip arthroscopy	0.1 % (1/1054 joints)	Unilateral	Traction damage	ND	Transient neurapraxia fully resolved within 6 h
Sampson [43]	Hip arthroscopy	0.2 % (1/530 joints)	Unilateral	Traction injury	ND	Complete recovery within 1 week (transient neurapraxia)
Andreani et al. [44]	Hip hemiarthroplasty	Case report	Unilateral	Nerve compression by iliopsoas haematoma	Severe sensitivity and motor deficit with persistent inguinal pain	Partial recovery after 6 months
Tokita et al. [45]	Internal fixation of an unstable intertrochanteric hip fracture in a patient with rheumatoid arthritis	Case report	Unilateral	Development of a haematoma in the hip joint after surgery, which was responsible for an increase in intra-articular pressure, leading to an enlargement of the iliopsoas bursa through the communication between the bursa and the hip joint	Exacerbation of a pre-existing femoral neuropathy secondary to iliopsoas bursitis: severe paraesthesia along the distal anterior part of the thigh and medial calf; quadriceps hyposthenia	Gradual resolution of symptoms after resection of the enlarged iliopsoas bursa
Ertem et al. [46]	Open reduction and innominate osteotomy with an anterior approach for the treatment of hip dysplasia	Case report	Unilateral	Stretching or accidental division of the nerve, mistaken for the psoas tendon	Difficulty walking; frequent falls; hyposthenia and hypotrophy of the quadriceps; abolished patellar reflex	6 years after the nerve graft required following the diagnosis of neurotmetic damage, almost complete recovery of motor function, but with residual quadriceps atrophy (15 months elapsed between hip dysplasia correction surgery and nerve repair)
Pedrotti et al. [47]	Renewal of a spica cast performed 6 weeks after closed reduction surgery under general anaesthesia with placement of a first spica cast in a 4 ½-month-old baby with hip dislocation	Case report	Unilateral	Nerve compression under the inguinal ligament due to prolonged cast immobilisation	Spontaneous limb hypomotility; evidence of discomfort/pain during passive mobilisation	Full recovery in 3 months
Kornbluth et al. [48]	Tourniquet assisted arthroscopic knee reconstruction	Case report	Unilateral	Nerve compression secondary to prolonged pneumatic tourniquet use	Quadriceps weakness; shin hypoesthesia	Almost complete recovery at 1 year (slight hyperesthesia above the medial malleolus and very slight hyposthenia of the quadriceps remained)
Mingo-Robinet et al. [49]	Tourniquet assisted surgical treatment of a patella fracture	Case report	Unilateral	Nerve compression secondary to prolonged pneumatic tourniquet use	Inability to extend the knee; moderate quadriceps atrophy; complete abolition of muscle strength	Very poor recovery at 18 months
Albrecht et al. [50]	Anterior cruciate ligament (ACL) reconstruction	Case series (17 cases)	Unilateral	Nerve damage secondary to prolonged use of pneumatic tourniquet or continuous femoral nerve block	Subjective weakness of the quadriceps; no recorded painful symptoms or paraesthesias	4 out of 17 patients (24 %) had clinical criteria or electrophysiological signs of femoral neuropathy at 4-week follow-up but not at 6-month follow-up
Diab et al. [51]	Spinal fusion and instrumentation for adolescent idiopathic scoliosis (AIS)	0.1 % (1/1301)	Unilateral	Direct peripheral compression	2/5 quadriceps femoris function (positional femoral neurapraxia)	Full recovery within 6 months
Papanastassiou et al. [52]	Extreme lateral interbody fusion (XLIF)	14.2 % (2/14)	Unilateral	Displacement of an endplate fragment	Psoas muscle spasm, quadriceps hyposthenia, and pain at the L2-L4 distribution level	Complete resolution after revision surgery
				Postoperative far-lateral herniation	Psoas and quadriceps muscle weakness with lower limb pain	
Almazrua et al. [53]	Extreme lateral interbody fusion (XLIF)	Case report	Unilateral	Nerve compression by a large iliopsoas muscle haematoma secondary to heparin anticoagulation (postoperatively) and antiplatelet therapy (preoperatively)	Left groin pain and numbness	Full resolution at 4 months
Ahmadian et al. [54]	Lateral retroperitoneal transpsoas lumbar interbody fusion	Case report	Unilateral	Excessive stretching of the femoral and obturator nerves	Weakness of iliopsoas, quadriceps, sartorius, and obturator muscles; burning paraesthesias of knee and thigh; anaesthesia of anterior and medial surface of the leg; intermittent neuropathic pain similar to shaking the whole limb	Good recovery of motor function at 1 year, despite stable and persistent sensory deficits
Robinson et al. [55]	Posterior spinal decompression	Case report	Unilateral	Nerve compression by iliopsoas haematoma	Postoperatively: mild groin pain; limitation of hip movement with pain on internal rotation. After 4 weeks: significantly worsened pain; right hip in forced flexion; marked painful limitation of hip movements; paraesthesias in the anterior and medial region of thigh and medial area of calf	6 weeks after haematoma evacuation, complete recovery of sensory-motor function but residual pain on extension and internal rotation of the hip
Kargel et al. [56]	Iliac crest bone harvest	Case series (2 cases)	Unilateral	Prolonged hip flexion or direct retractor injury	Numbness and weakness in the lower limb; abolition of the patellar reflex	Full recovery within 6 months
				Nerve compression by a retroperitoneal haematoma	Weakness of thigh and leg flexion; reduced leg sensitivity	
Farrow et al. [57]	Iliac crest bone harvest with postoperative infusion of bupivacaine via catheter at the bone graft harvest site	Case report	Unilateral	Anaesthetic diffusion towards the femoral nerve	Numbness in thigh and calf; severe quadriceps hyposthenia; abolished patellar reflex; hypoesthesia in anteromedial thigh region	Complete resolution in 24 h
Toro et al. [58]	Harvesting of a microvascular iliac flap for mandibular reconstruction	Case report	Unilateral	Nerve compression by iliacus muscle haematoma secondary to heparin anticoagulation	Forced position of the limb (hip flexion, knee flexion, external rotation of the hip); loss of sensitivity at the level of the L2-L3-L4 dermatomes; abolition of the knee jerk reflex; impossibility of active knee extension	Almost total recovery within 6 months
Tonetti et al. [59]	Anterolateral extraperitoneal exposure to the anterior lumbar spinal column	Case series (3 cases)	Unilateral	Nerve stretching when the surgeon retracted the psoas muscle to expose the lateral side of the vertebral body	Numbness in the front part of the thigh; quadriceps hyposthenia; patellar reflex abolished (same clinical situation in all 3 cases)	Partial recovery after 12–18 months (persistence of sensory disturbances in the thigh and abolition of the patellar reflex)

ND: Not described in the paper.

**Table 4:** Reports of iatrogenic femoral nerve injury in abdominal surgery (2000–2021).

Reference	Procedure	Frequency	Uni/bilateral	Probable pathogenesis	Clinical presentation	Outcome
Bono et al. [60]	Hartmann's procedure	Case report	Bilateral	Nerve compression by the retractor	Bilateral weakness of the knee extensor and hip flexor, as well as iliopsoas and quadriceps; hypoesthesia of the anteromedial surface of the thigh; bilateral patellar areflexia	Persistent neuropathy after 1 year
Thoms et al. [61]	Hartmann's procedure	Case report	Unilateral	ND	Mild atrophy with hypotonia of the limb; marked hyposthenia in knee extension; active quadriceps contraction abolished; mild hypoesthesia at the medial surface of the calf	Full recovery within 3 years
Barrett et al. [62]	Right hemicolectomy	Case report	Unilateral	Complete transection of the nerve	Inability to extend the knee; severe pain with anaesthesia in the femoral nerve sensory territory	Incomplete but satisfactory recovery 36 months after reconstruction by bridging with reversed interpositional sural nerve cable grafts
Kuo et al. [63]	Subtotal colectomy with ileorectal anastomosis	Case report	Unilateral	Ischaemia of the nerve trunk secondary to compression by the inguinal ligament, which is strained due to the lithotomy position	Numbness and weakness of the thigh; hypoesthesia of the knee and medial surface of the thigh; hyposthenia of the hip flexors and knee extensors	Complete recovery by 3 months
Kuo et al. [63]	Abdominoperineal resection	Case report	Unilateral	Ischaemia of the nerve trunk secondary to compression by the inguinal ligament, which is strained due to the lithotomy position	Numbness in the thigh; weakness in the lower leg; hypoesthesia in the medial aspect of the thigh; slight hyposthenia of the hip flexors and knee extensors	Persistence of mild paraesthesia of the thigh at 3 months
Huang et al. [64]	Proctectomy with colo-J-pouch anal anastomosis	Case report	Unilateral	Nerve compression by the self-retaining retractor system	Pain when lying down; difficulty in flexing the hip and extending the knee; numbness and paraesthesias at the front of the thigh; hyposthenia of the hip flexors and knee extensors	Motor recovery after 2 months; sensory recovery after 6 months
Huang et al. [64]	High anterior resection of colonic cancer with colorectostomy	Case report	Bilateral	Nerve compression by the self-retaining retractor system	Bilateral weakness of the knee extensors with relatively preserved strength of the hip and ankle flexors and extensors; hypoesthesia in the medial region of the thighs	Motor recovery after 6 months; sensory recovery after 15 months
Huang et al. [64]	Reversal of Hartmann's procedure	Case report	Unilateral	Nerve compression by the self-retaining retractor system	Inability to extend the leg; paraesthesias in the anterior quadrant of the thigh; weakness of both the left knee extensor and left hip flexor	Almost complete recovery after 9 months
Huang et al. [64]	Anterior resection with colorectal anastomosis and sacral fixation for rectal procedentia	Case report	Unilateral	Nerve compression by the self-retaining retractor system	Numbness with tingling sensation in the medial surface of the thigh; hyposthenia of the thigh in hip flexion and knee extension; hypovalid patellar reflex	Full recovery at 6 months
Ducic et al. [65]	Colostomy reversal	Case report	Unilateral	Nerve compression by the self-retaining retractor system, combined with the lithotomy position held by the patient during surgery	Inability to extend the leg	Almost complete recovery at 1 year after external neurolysis
Kell and O'Connell [66]	Abdominoperineal resection	Case report	Unilateral	Direct compression of the nerve by the elbow of one of the surgeons	ND	Full recovery within 6 weeks
Celebrezze et al. [67]	Abdominoperineal resection	Case report	Unilateral	Nerve compression by ring-type self-retaining retractor	Numbness in the thigh; hyposthenia in hip flexion and leg extension	Full motor recovery within 2 months (moderate numbness in the thigh remained)
Celebrezze et al. [67]	Low anterior resection (LAR)	Case series (2 cases)	Unilateral	Nerve compression by ring-type self-retaining retractor	Numbness of the thigh; hyposthenia of the left leg when walking; limited knee extension	50 % recovery of strength 6 months after surgery
					Weakness in leg extension; numbness of lateral thigh surface	Full motor recovery but with residual numbness in the thigh
Brown and Shorthouse [68]	Abdominal rectopexy with birch colposuspension	Case report	Unilateral	Nerve compression by the retractor	Paraesthesia in the L2-L3 distribution; quadriceps hyposthenia	Almost full recovery after neurolysis treatment
Kim et al. [10]	Appendectomy	Case series (2 cases)	Unilateral	ND	ND	Good recovery in one of the two patients, who underwent neurolysis. Functional recovery to grades 4 and 5 according to LSUHSC muscle grading system within 2 years after graft repair for the other patient
Kim et al. [10]	Hernioplasty reoperation for complications of the first intervention (haematomas, infections, recurrent hernias)	Case series (10 cases)	Unilateral	ND	Severe motor impairment; excruciating pain in 7 out of 10 patients	Grade 3 or better recovery according to LSUHSC muscle grading system in all 10 cases (7 treated with neurolysis, 2 with suture repair, 1 with graft repair)
Azuelos et al. [69]	Laparoscopic inguinal hernia repair	Case series (5 cases)	Unilateral	ND	ND	Full recovery after neurolysis treatment
Lange et al. [70]	Totally extraperitoneal laparoscopic inguinal hernia repair	Case report	Unilateral	Anatomical constriction due to post-surgical inflammatory oedema; spontaneous genesis not excluded	Numbness in the medial side of the leg; limb weakness; tendency to fall	Partial recovery after surgical nerve revision
García-Ureña et al. [71]	Mesh hernioplasty for a re-recurrent inguinal hernia	Case report	Unilateral	Intraoperative manipulation/compression by the inguinal ligament newly constructed with the mesh	Severe thigh pain; inability to walk; leg weakness; tendency to fall	Full recovery after 6 months
Dubuisson et al. [72]	Hernioplasty for recurrent inguinal hernia	Case report	Unilateral	ND	Severe thigh pain with quadriceps paralysis	Good recovery 4.5 years after nerve graft reconstruction following diagnosis of nerve trunk transection
Sharma et al. [73]	Kidney transplantation	2.2 % (4/184)	Unilateral	Nerve compression from prolonged use of self-retaining retractors	Severe weakness in hip flexion and knee extension; no knee jerk; hypoesthesia on anteromedial side of thigh and medial side of leg	Complete motor recovery after 6 months; residual thigh hypoesthesia
						Complete motor recovery after 9 months; residual thigh hypoesthesia
						Complete motor recovery after 4 months; residual feet hypoesthesia
						Complete motor and sensitive recovery after 6 months
Yamada et al. [74]	Kidney transplantation	Case report	Unilateral	Incorrect positioning of retractor associated with omitted releasing of it while waiting for donor kidney	Inability to straighten limb; quadriceps paresis; reduced thigh sensitivity	Complete recovery of motor function within 6 months, but persistence of neuropathic pain
Van Veer et al. [75]	Kidney transplantation	0.14 % (5/3448)	Unilateral	Direct compression of the nerve and interruption of blood supply	Weakness of the quadriceps muscle; absent knee jerk	Complete recovery by 3 months
			Unilateral		Paresis of the iliopsoas muscle and the quadriceps muscle; absent knee jerk	Full recovery by 1 year
			Unilateral		EMG showing poor contraction in the medial and lateral vastus muscle	Patient dead 2 months after transplantation
			Unilateral		Weakness of muscle strength in the thigh	Incomplete recovery
			Unilateral		Paresis of the iliopsoas muscle	Complete recovery within 3 months
Kim et al. [76]	Kidney transplantation	Case series (5 cases)	Unilateral	Mechanical injury	Numbness of the thigh associated with weakened hip flexion and knee extension	Full recovery after 2 months
			Unilateral	Ischaemic injury		Full recovery after 2 months
			Unilateral	Mechanical injury		Full recovery after 2 months
			Unilateral	Mechanical injury		Full recovery within 2 weeks
			Unilateral	Mechanical injury		Full recovery within 2 weeks
Nikoobakht et al. [77]	Kidney transplantation	3.36 % (4/119)	Unilateral	Ischaemic damage due to occlusion of vessels to the nerve; direct compression of the nerve by the transplanted kidney, a retractor or a haematoma	Muscle paresis in all 4 patients; pain and hyperesthesia in all patients; paraesthesia in 2 out of 4 patients	ND
Kesikburun et al. [78]	Nephrectomy	Case report	Unilateral	Stretching of the nerve during operation	Numbness, tingling and muscle weakness	Partial recovery after 6 months
Ashraf et al. [79]	Percutaneous Simple Renal Cyst Sclerotherapy with Ethanol	Case report	Unilateral	Rupture of the cyst with leakage of ethanol, resulting in direct nerve damage	Burning pain, lameness, numbness and weakness in the anterolateral surface of the thigh	Lack of recovery of nerve function (the sensory component was most affected)
Azuelos et al. [69]	Intra-abdominal vascular surgery	Case series (2 cases)	Unilateral	ND	ND	Full recovery after neurolysis treatment
Kim et al. [10]	Lumbar sympathectomy	Case report	Unilateral	L3 spinal nerve transection	Full motor deficit	Motor function grade 4 according to LSUHSC muscle grading system 2 years after nerve grafting surgery

ND: Not described in the paper.

**Table 5:** Reports of iatrogenic femoral nerve injury in gynaecological surgery (2000–2021).

Reference	Procedure	Frequency	Uni/bilateral	Probable pathogenesis	Clinical presentation	Outcome
Cardosi et al. [80]	Major gynaecological oncologic surgery	0.25 % (3/1210)	Unilateral	Compression by retractor blade	Lower extremity weakness (2 patients); paraesthesia (1 patient)	Complete resolution in 3 weeks, 6 months and just under 9 months respectively
Talaván-Serna et al. [81]	Abdominal hysterectomy	Case report	Unilateral	Direct compression by surgical valves	Hypoesthesia in the anterior thigh, quadriceps paresis and abolition of the patellar reflex	Full recovery within 6 months
Celebrezze et al. [67]	Abdominal hysterectomy with sigmoid colectomy for a colouterine fistula	Case report	Unilateral	Nerve compression by ring-type self-retaining retractor	Hyposthenia of the iliopsoas and quadriceps muscles; no sensory deficits	Almost full recovery within 6 months
Gupta et al. [82]	Vaginal hysterectomy	Case series (2 cases)	Bilateral	Local nerve trunk ischaemia secondary to reduced blood supply due to compression of the stretched inguinal ligament secondary to the lithotomy position	Bilateral numbness of thighs and knees; inability to walk; hypoesthesia at the front of the thighs and around the knee joint; hypoesthesia of the hip flexors and knee extensors; knee jerk absent	Almost complete recovery by 15th postoperative day (minimal numbness remained)
					Bilateral anteromedial thigh numbness; inability to walk; hyposthenia of hip flexors and knee extensors; abolished patellar reflex	Full recovery by 7th postoperative day
Bal et al. [83]	Vaginal hysterectomy	Case series (3 cases)	Bilateral	Nerve entrapment at the inguinal ligament due to the lithotomy position	Severe quadriceps hyposthenia (more on the right side); no knee jerks; numbness on the anterior aspect of both thighs	Full recovery within 6 weeks
			Unilateral		tingling and numbness of the thigh; hypoesthesia of the anteromedial aspect of the thigh and medial side of the calf	Full recovery within 1 week
			Unilateral		Sensorimotor deficits	Patient lost to follow-up
Baxi et al. [84]	Vaginal hysterectomy	Case series (2 cases)	Unilateral	Nerve ischaemia secondary to compression by the inguinal ligament caused by the lithotomy position	Tendency to fall secondary to buckling of the knee; difficulty climbing stairs	Full recovery within 8–10 weeks
Porzionato et al. [85]	Laparoscopic ovariectomy	Case report	Unilateral	Direct nerve injury caused by trocar insertion	Thigh weakness and allodynic paraesthesias	Irreversible neuropathy
Kumar et al. [86]	Open oophorectomy	Case report	Unilateral	Compression by self-retaining retractor	Pain and numbness in the anterior and medial region of the thigh extending to the knee; tendency to fall	Almost complete recovery after 8 months
Watanabe et al. [87]	Radical ovarian cancer surgery	Case report	Unilateral	Inguinal ligament compression secondary to self-retaining retractor use and lithotomy position	Difficulty in knee extension; paraesthesia of the lower limb	Full recovery after 20 months
Maneschi et al. [88]	Abdominal surgery for gynaecologic cancer	2.23 % (12/538)	Unilateral	Nerve compression by Bookwalter retractor	Quadriceps weakness in 11 patients (8 with an MRC score of 0–1; 2 with an MRC score of 2–3; 1 with a score of 4); hyposthenia of the quadriceps, hip flexor and pectineus with MRC of 2 out of 3, patellar areflexia and sensitivity deficit of the anteromedial area of the thigh in the twelfth patient	Median recovery time 70 days (range 30–120); full motor recovery in 9 cases (3 patients still had mild hyposthenia, with MRC 4/5); all 12 patients still had a sensory deficit in the anteromedial area of the thigh
Kim et al. [10]	Resections of endometriosis-associated cysts; salpingo-oophorectomies; hysterectomies	Case series (8 cases)	Unilateral	Retraction-induced stretch and compression	Severe motor impairment (in 2 cases grade 0 according to the LSUHSC muscle grading system and in 6 cases grade between 0 and 2)	Good recovery in all patients, 6 of them treated with neurolysis and 2 with graft repairs
Bohrer et al. [89]	Elective gynaecological surgery for benign or malignant conditions (vaginal and laparoscopic surgery)	0.5 % (3/616)	2 bilateral 1 unilateral	Stretching of the nerve due to its sharp angle, attributable to the lithotomy position	1 purely sensory bilateral neuropathy; 1 bilateral sensorimotor neuropathy; 1 purely sensory unilateral neuropathy with anaesthesia and paraesthesia	Recovery time between 112 and 298 days for the two bilateral neuropathies; persistence of symptoms at 8-month follow-up for the unilateral neuropathy

ND: Not described in the paper.

**Table 6:** Reports of iatrogenic femoral nerve injury in urologic surgery (2000–2021).

Reference	Procedure	Frequency	Uni/bilateral	Probable pathogenesis	Clinical presentation	Outcome
Castrillo et al. [90]	Prostatectomy	Case report	Bilateral	Nerve compression by a retractor	Hypotonia of both knee extensors; decreased strength of muscles of both lower limbs; decreased bilateral patellar reflex	Almost complete recovery within 4 months after surgery
Noldus et al. [91]	Ureteroneocystostomy	Case report	Unilateral	Compression by a self-retaining retractor	Numbness and severe thigh weakness	Full recovery
Corbu et al. [92]	Augmentation ileocystoplasty	Case report	Unilateral	Compression by a self-retaining retractor	Severe quadriceps hyposthenia; hypoesthesia along the anteromedial surface of the thigh	Full recovery after 3 months
Corbu et al. [92]	Radical cystoprostatectomy and reconstruction by orthotopic ileal neobladder	Case report	Bilateral	Compression by a self-retaining retractor	Bilateral hyposthenia of the hip flexor muscles; bilateral anaesthesia of the anteromedial surface of the thighs; patellar reflex absent bilaterally	Full recovery within 3 months but subsequent development of unilateral quadriceps hypotrophy
Pinto et al. [93]	Psoas hitch vesicopexy in ureteral reimplantation	Case series (2 cases)	Unilateral	Incorrect positioning of retractors	Weakness in the left leg; difficulty walking and inability to climb stairs; hyposthenia in hip flexion and knee extension; decreased tactile sensation in the anteromedial region of the thigh	Full recovery within 1 year
					Significant leg weakness; impossibility to climb stairs; decreased tactile sensation of the anterior-medial region of the thigh and medial area of the leg	Almost full recovery within 1 year (residual difficulties in climbing stairs)
Müller et al. [94]	Open retroperitoneal distal ureterectomy followed by a ureteroneocystostomy with a vesico-psoas hitch	Case report	Unilateral	Mechanical nerve damage caused by the sutures used to hitch the bladder to the psoas muscle	Excruciating pain, hypoesthesia and weakness in the anterior segment of the leg, exacerbated on palpation, hip flexion, knee extension and leg exorotation	Mild signs of lower limb weakness 4 months after re-exploration surgery

ND: Not described in the paper.

**Table 7:** Reports of iatrogenic femoral nerve injury in endovascular procedures and vascular surgery (2000–2021).

Reference	Procedure	Frequency	Uni/bilateral	Probable pathogenesis	Clinical presentation	Outcome
Kim et al. [10]	Transfemoral percutaneous catheterisation for angiography	Case series (7 cases)	Unilateral	Compression of the nerve by haematomas or pseudoaneurysms at puncture sites	Motor deficits associated in some cases with very severe pain syndromes	Good motor recovery in all patients, who were treated with neurolysis, which led to only partial resolution of pain
Desai et al. [95]	Intravenous puncture of the femoral vein for angiography or for blood sample collection procedure	Case series (4 cases)	Unilateral	Penetrating injury of the nerve by the needle	ND	Complete functional recovery in the 3 patients undergone external neurolysis; no recovery in the patient undergone nerve grafting
Azuelos et al. [69]	Transfemoral percutaneous catheterisation for coronary angiography	Case series (3 cases)	Unilateral	ND	ND	Full recovery after neurolysis treatment
Özkan et al. [96]	Transfemoral percutaneous catheterisation for coronary angiography	Case report	Unilateral	Nerve compression due to sandbag application in the groin region	Buckling of the knee during walking; mild quadriceps atrophy diminished sensation on the left anterior thigh and medial calf; absent knee jerk	Full recovery within 3 months
Barçın et al. [97]	Transfemoral percutaneous catheterisation for coronary angiography	Case series (2 cases)	Unilateral	Accumulation of local anaesthetic drug injected before the catheterisation procedure around the femoral artery or injection of the drug inside the myelin sheath of the nerve	Numbness in the thigh	Full recovery within 18 h
					Severe quadriceps weakness with anaesthesia in the anterior and medial region of the thigh	Full recovery within 24 h
Hsin et al. [98]	Transfemoral percutaneous catheterisation for intra-aortic balloon pump (IABP) positioning	Case report	Unilateral	Direct nerve injury due to excessive outer diameter of IABP catheter introducer sheath (9.5-Fr)	Motor weakness of the thigh; mild sensory disturbance	Full recovery after 6 months
Baba et al. [99]	Transfemoral percutaneous catheterisation for leadless pacemaker positioning	Case report	Unilateral	Compression and stretching of the nerve by the large-bore delivery sheath used during femoral venous access	Intense pain at the level of the antero-lateral part of the thigh during the progression of the delivery sheath; no sensory or motor deficits	Pain disappeared on sheath withdrawn
El Ghanem et al. [100]	Transfemoral percutaneous catheterisation	0.0038 % (597/15,894,201), that is 3.8 per 100,000 procedures	Unilateral	ND	ND	ND
Jang et al. [101]	Insertion of femoral cannulae for extracorporeal membrane oxygenation therapy (ECMO)	Case report	Unilateral	Nerve compression due to the presence of the inserted cannulas at the femoral level as well as diffuse tissue oedema secondary to a post-thrombotic syndrome following deep vein thrombosis	Impaired function, pain, and hyperesthesia of the LE	Partial recovery after 6 months (remained tingling sensation)
Ginanneschi et al. [102]	Crossectomy and stripping of the great saphenous vein	Case report	Unilateral	Damage to intermediate and medial femoral-cutaneous nerves (cutaneous branches of the femoral nerve) as a result of the multiple stab avulsion procedure (which consists of making several tiny skin incisions) performed on completion of saphenous stripping	Sensory loss in the lower two-thirds of the antero-medial surface of the thigh; no motor deficits	ND
Öztürk et al. [103]	PTA of lower limb arteries	Case report	Unilateral	Nerve compression due to bladder distention	Inability to flex thigh	Transient with full recovery
Kim et al. [10]	Aortofemoral bypass intervention	Case series (8 cases)	Unilateral	Nerve compression due to a haematoma, a pseudoaneurysm or a haematoma caused by the rupture of a pseudoaneurysm	Almost complete motor deficit in all patients, in some cases associated with severe pain syndromes	Good recovery in all patients (3 treated with neurolysis, 5 with graft repair)

ND: Not described in the paper.

**Table 8:** Reports of iatrogenic femoral nerve injury in anaesthesiological procedures (2000–2021).

Reference	Procedure	Frequency	Uni/bilateral	Probable pathogenesis	Clinical presentation	Outcome
Olsen et al. [104]	Non-ultrasound-guided ilioinguinal field block with liposomal bupivacaine for inguinal herniorrhaphy	Case report	Unilateral	Diffusion of the anaesthetic towards the femoral nerve due on the one hand to the blind approach of the injection and on the other hand to the large volume of liposomal bupivacaine injected (20 mL)	Inability to walk due to limb weakness; numbness in anterolateral thigh region and medial surface of entire lower limb; knee extension against gravity prevented	Full sensory recovery after 45 h; full motor recovery at 51 h
Auroy et al. [105]	Single shot femoral nerve block	0.03 % (3/10,309)	Unilateral	ND	ND	ND
Ducic et al. [65]	Single shot femoral nerve block	Case report	Unilateral	ND	Knee extension abolished	Partial recovery 6 months after nerve decompression and internal neurolysis
Tsai et al. [106]	Single shot ilioinguinal nerve block	Case report	Unilateral	Anaesthetic diffusion towards the femoral nerve due to anatomical continuity between the transversalis and iliacus fascia	Quadriceps weakness; sensory loss over the anterior thigh	Full recovery at 8 h after surgery (transient femoral nerve palsy)
Ghani et al. [107]	Single shot ilioinguinal nerve block	5 % (10/200)	Unilateral	Anaesthetic diffusion towards the femoral nerve due to anatomical continuity between the transversalis and iliacus fascia	Sensory loss on the anterior aspect of the thigh; weakness in knee extension	Full recovery within 24 h in all cases
Udo et al. [108]	Single shot ilioinguinal nerve block	2.6 % (3/112)	Unilateral	Anaesthetic diffusion towards the femoral nerve due to anatomical continuity between the transversalis and iliacus fascia	Sensory loss in the anterior part of the thigh; quadriceps hyposthenia	Full recovery within 2–6 h
Schafhalter-Zoppoth et al. [109]	Single shot femoral nerve block	Case report	Unilateral	Inadvertent femoral nerve impalement with subtotal intra-neural injection	Transient loss of sensation with no motor disturbances	Full spontaneous recovery within 48 h
Cuvillon et al. [110]	Postoperative continuous femoral nerve block	0.5 % (1/211)	Unilateral	ND	Femoral paraesthesia	Partial recovery after 1 year
Capdevila et al. [111]	Postoperative continuous femoral nerve block	0.4 % (3/683)	Unilateral	ND	ND	Complete recovery within 10 weeks
Maeda et al. [112]	Postoperative continuous femoral nerve block	Case report	Unilateral	Presence of a distal kink preventing the removal of the catheter	Severe neuralgic pain during catheter removal procedures	Total disappearance of pain after surgical removal of the catheter, that was cut in two parts so it could be removed
Feibel et al. [113]	Postoperative continuous femoral nerve block	0.8 % (9/1190)	Unilateral	ND	ND	In 7 cases the symptoms resolved within 3 months; in 2 patients, the persistence of a quadriceps strength deficit (4/5 MRC scale) and a sensitivity deficit was observed
Blumenthal et al. [114]	Postoperative continuous femoral nerve block	Case report	Unilateral	Anaesthetic neurotoxicity due to marked susceptibility to toxic nerve damage from a pre-existing neuropathy	Persistent quadriceps weakness; hyposensitivity in medial aspect of thigh limited to L3 dermatome; patellar reflex abolished	Full recovery after 6 months
Manatakis et al. [115]	Transversus abdominis plane (TAP) block	Case series (2 cases)	Unilateral	Incorrect injection site (between transversus abdominis muscle and transversalis fascia), resulting in accumulation of anaesthetic around the femoral nerve	Inability to extend the knee; quadriceps paresis; hypoesthesia on anterior aspect of thigh; patellar reflex abolished	Complete resolution within 24 h
					Quadriceps muscle weakness; hypoesthesia of the anterior thigh	Complete resolution within 8 h
Mellert et al. [116]	Postoperative ilioinguinal-iliohypogastric nerve blocks (II/IH-NB)	Case report	Unilateral	Anaesthetic diffusion towards the femoral nerve due to anatomical continuity between the transversalis and iliacus fascia	Numbness in the anterior part of the thigh; hip flexor hyposthenia; quadriceps paralysis; inability to walk	Complete recovery of neurologic function 8 h postoperatively (self-limited femoral nerve palsy)
Al Nasser and Palacios [117]	Continuous psoas compartment block	Case report	Unilateral	Direct needle trauma to nerve roots	Loss of sensory and motor nerve function	Full recovery within 6 months
Güngör et al. [118]	Lumbar plexus blockade (LPB)	Case report	Unilateral	Accidental intraneural-intrafascicular injection with myelinic and axonal degeneration	Leg weakness; reduced knee extension; marked quadriceps atrophy	Almost complete recovery within 6 months
Haber et al. [119]	Postoperative intramuscular meperidine injection	Case series (2 cases)	Unilateral	Accidental intraneural-intrafascicular injection with myelinic and axonal degeneration	Atrophy of the vastus lateralis and vastus medialis; leg weakness; burning pain in the injection area	Partial recovery at 2-month follow-up
					Hyperesthesia on the lateral aspect of the thigh; distal lateral thigh atrophy; worsening pain in the injection area	Almost complete recovery at 10 months

ND: Not described in the paper.

**Table 9:** Reports of iatrogenic femoral nerve injury in plastic surgery (2000–2021).

Reference	Procedure	Frequency	Uni/bilateral	Probable pathogenesis	Clinical presentation	Outcome
Kirby [120]	Medial thigh lift in a formerly obese patient who lost 30 kg	Case report	Unilateral	Nerve compression by a large post-surgical haematoma	Pain and paraesthesia in the thigh; inability to move the leg (due to pain and weakness); hip in forced external rotation, with inability to adduct the thigh and extend the knee	Complete resolution within 24 h
Pechter and Smith [121]	Abdominoplasty	Case report	Unilateral	Intraoperative compression, suture ligation or side effect of the local anaesthetic infiltrated into the incision site	Numbness and loss of strength in the thigh; inability to extend the knee; reduced patellar reflex; anaesthesia in the anteromedial thigh and medial leg	Full recovery within 48 h

## 3 Results

We identified 104 articles on iatrogenic femoral nerve injury, which we outline in Table 3, Table 4, Table 5, Table 6, Table 7, Table 8, Table 9.

Each table corresponds to each of the seven surgical contexts of the reviewed articles.

Of the 104 papers reviewed, 78 were case reports or case series, while 26 were retrospective analyses.

**Fig. 1:**
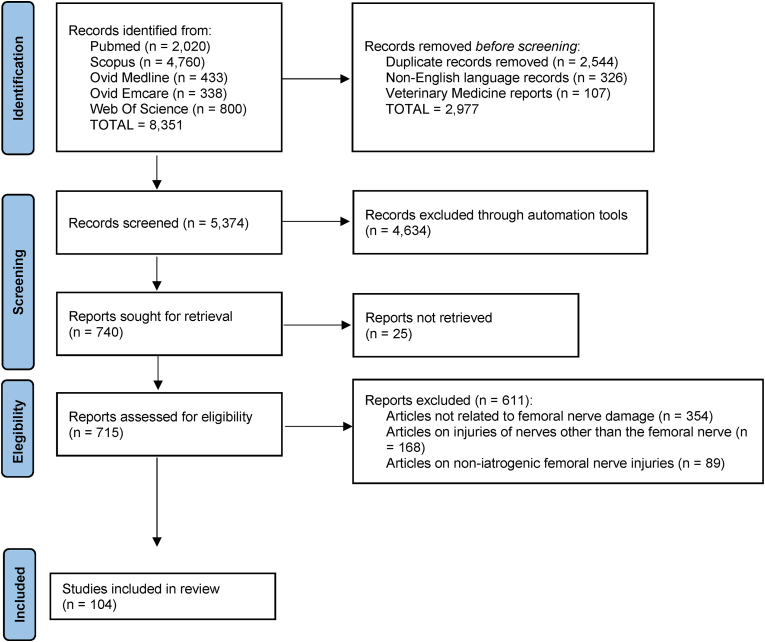
PRISMA-ScR (Preferred Reporting Items for Systematic Reviews and Meta-Analyses extension for Scoping Reviews) flow diagram for study selection.

We used the Preferred Reporting Items for Systematic Reviews and Meta-Analyses extension for Scoping Reviews (PRISMA-ScR) flow diagram guidance [17] to depict the information flow through the several phases of this Scoping Review (Fig. 1).

The main limitations of this Scoping Review are that it did not consider the grey literature and based the search strategy on only 5 databases.

The limitation of the review procedure was the use, in the first phase (initial screening phase), of an automation system, which could reasonably have eliminated some articles suitable for the purposes of the review.

## 4 Discussion

This section presents a concise discursive overview that illustrates the main features of femoral nerve injuries in the various surgical branches.

A summary of the clinical and prognostic features of the lesion is then proposed, as well as a review of the main emerging preventive strategies identified in the literature examined.

Finally, the main medico-legal implications related to the topic under discussion are discussed.

### 4.1 Iatrogenic femoral nerve injury in orthopaedic surgery

The frequency of peripheral nerve lesions during total hip arthroplasty ranges from 0.3 % to 3.7 % [122,123].

The most commonly damaged nerve, with a reported frequency of between 0.05 % and 1.9 %, is the sciatic nerve [124].

With particular reference to lesions of the femoral nerve, according to the largest retrospective studies, the incidence of this complication after THA is between 0.01 % and 0.26 % [25,26].

The main mechanisms of damage are the following: excessive lengthening of the limb; nerve compression by self-retaining retractors, haematomas, or pseudotumours; damage due to extruded methylmethacrylate from cemented prostheses (by compression or heat secondary to the exothermic reaction of polymerisation); and laceration from a screw used in the acetabular component. However, only in rare cases is the exact aetiology of the nerve lesion clearly identifiable [125].

The surgical procedure can be performed through three approaches: posterior, direct lateral, and direct anterior.

According to several studies, the direct anterior approach, although characterised by various advantages (shorter rehabilitation, quicker resumption of daily activities, reduced postoperative pain, less obvious surgical scar, and less blood loss), is reported to be accompanied by higher complication rates, particularly during the so-called “learning curve period” for the surgeon.

In a recent retrospective analysis, Hoshino et al. [34] examined 1059 primary THAs (160 bilateral and 739 unilateral) performed between 2007 and 2014 at their institution.

The direct anterior approach was used in 273 cases, the anterolateral approach in 126 cases, and the posterolateral approach in 660 cases.

The reported incidence of iatrogenic femoral nerve injury with direct anterior approach was 1.1 % (3 cases out of 273 operated joints), which is consistent with previous studies investigating the frequency of femoral nerve injury in THA with direct anterior approach [26,33] and not significantly higher than the frequency reported for other approaches.

Accidental femoral nerve injury during hip arthroscopy is reported with a frequency of 0.1–0.2 % [42,43].

The damage mechanism that occurs during the procedure is most likely a distractive one related to the prolonged joint traction required for proper visualisation of the innermost regions of the hip socket [43].

In knee surgery, several cases of femoral nerve damage caused by the prolonged use of pneumatic tourniquet, a device that has been widely used for more than 60 years because of its ability to prevent bleeding and technical impediment [[48], [49], [50]].

The device is placed at thigh level with an inflation pressure of around 300 mmHg for a maximum recommended 3 h [126].

Excessive inflation pressure (above 350 mmHg) and too long a duration of tourniquet hold were shown to be associated with an increased risk of neuroapraxic compression injury [127].

An approximately three-fold increase in the risk of neurological complications was demonstrated for every 30 min of prolonged device retention [128].

Interestingly, while this is a pure compression injury, in one case an unfavourable evolution was reported, with limited recovery of nerve function at 18 months [49].

In the case of spinal surgery, several cases of femoral nerve injury following lumbar arthrodesis operations are reported. In such operations, the branches of the lumbar plexus are particularly at risk of damage because of anatomical continuity.

Although the role of intraoperative EMG monitoring is indispensable, its diagnostic ability might be overestimated, as plexic lesions are mostly multifactorial [54].

Several possible mechanisms of damage are described: excessive stretching, direct compression or compression by haematoma of the psoas, displacement of an endplate fragment, and postoperative herniation [[51], [52], [53], [54], [55]].

Finally, some cases of femoral nerve injury following iliac crest bone harvesting are reported. In these cases, the cause of the nerve injury was found to be compression due to retroperitoneal haematoma, excessive traction, or diffusion of anaesthetic [[56], [57], [58]].

### 4.2 Iatrogenic femoral nerve injury in abdominal surgery

In the case of colorectal surgery, in most reported cases, the nerve damage is compressive and related to the use of self-retaining retractors, which may result in constriction of the nerve against the abdominal wall (on its way into the psoas muscle) or the lateral pelvic wall.

The self-retaining retractors most likely to compress the nerve trunk are the so-called “ring retractors”, which are often used because they allow multidirectional exposure of the surgical site [67].

Indirect compressive damage is also possible, in other words, primarily ischaemic damage secondary to compression of the iliac vessels.

In a reported case, the compressive effect was not exerted by a surgical retractor but by the direct pressure exerted by the elbow of one of the surgeons during a complex abdominoperineal resection operation [66].

In inguinal hernia repair surgery, the most common mechanisms of purely surgical nerve damage (excluding anaesthetic procedures) are accidental suturing of the nerve, entrapment within scar tissue, and compression by the action of direct intraoperative pressure or postoperative oedematous tissue [71,72]. This complication occurs most frequently in operations for recurrent hernias, evacuation of haematomas, or debridement of surgical infections [129].

Iatrogenic femoral nerve injury has also been described during laparoscopic inguinal hernia repair [130].

The incidence of femoral nerve injury after renal transplantation ranges from 0.14 % to 8.4 % [75,76,131,132].

Three pathogenetic mechanisms are noted: physical compression, traction or impact damage, and ischaemic injury

In the case of compressive etiopathogenesis, an anatomical feature that makes the femoral nerve vulnerable to compression damage by surgical retractors during renal transplantation is the exceptionally shallow position it occupies within a groove between the iliacus and psoas major muscles.

However, the compressive effect may be secondary to the formation of a perineural haematoma intraoperatively or postoperatively, which is particularly common in individuals on anticoagulant/antiplatelet therapy or with haemocoagulation disorders.

Furthermore, ischaemic damage may occur because of a temporary ischaemic period of the nerve secondary as a result of clamping the iliac artery. This ischaemic period, which is usually relatively short, may, however, result in considerable ischaemic damage in patients with circulatory disorders (e.g., diabetes mellitus) and because of the relatively poor vascularisation of the femoral nerve at the pelvic level, especially on the left side [7,75].

Moreover, in renal surgery, cases of nerve damage are reported during nephrectomy (due to excessive nerve stretching) [78] and percutaneous sclerotherapy of renal cysts (due to toxic damage from ethanol leakage) [79].

Finally, a single case of transection of the L3 spinal root during sympathectomy is reported, with neurotmesic damage treated by nerve grafting with good recovery of function at 2 years [10].

### 4.3 Iatrogenic femoral nerve injury in gynaecological surgery

According to the two most authoritative retrospective studies on the subject, the incidence of iatrogenic femoral nerve injury during major gynaecological oncological procedures is between 1.1 % and 1.9 % [80,133]. However, at present, the incidence of this nerve injury in the context of routine gynaecological surgery has not been precisely defined.

The gynaecological operation most frequently associated with the complication appears to be hysterectomy, with a reported post-surgical incidence of between 7.45 % and 11.6 % [134,135].

Two pathophysiological mechanisms are most commonly responsible for nerve damage during hysterectomy: stretching and compression of the nerve against the inguinal ligament secondary to the lithotomy position (in this position, the hip is flexed, adducted, and externally rotated, thus giving the nerve a particularly sharp angle) in the case of transvaginal procedure, and compression of the nerve by retractors in the case of abdominal hysterectomy.

Femoral nerve injury during ovariectomy is also reported and is generally secondary to the improper use of retractors [10,86,87]; ]; a case is also reported in which nerve damage was caused by an incorrect trocar insertion procedure during laparoscopic surgery [85 Nevertheless, in gynaecological surgery, bilateral nerve damage is not uncommon [136].

### 4.4 Iatrogenic femoral nerve injury in urologic surgery

The pathogenetic mechanism most commonly associated with femoral nerve injury during urological procedures is compression of the nerve by self-locking retractors, which are typically held in the pelvis for prolonged periods during complex procedures such as radical cystectomies or prostatectomies.

Less frequently, three further damage mechanisms are involved: direct damage due to accidental cutting or coagulation, nerve ischaemia due to interruption of the intrapelvic blood flow, and nerve traction in a lithotomy position with hip hyperabduction [137]. In urological surgery, as in gynaecological surgery, bilateral involvement of the femoral nerve is also possible.

### 4.5 Iatrogenic femoral nerve injury in endovascular procedures and vascular surgery

Another typical context in which iatrogenic lesions of the femoral nerve can occur is femoral vessel catheterisation procedures (for blood sampling, angiographic procedures, intra-aortic balloon pump placement, leadless pacemaker insertion, and extracorporeal membrane oxygenation).

According to a recent retrospective study conducted by El-Ghanem et al. on 15,894,201 percutaneous catheterisation procedures, the incidence of this complication is 3.8 events per 100,000 operations [100].

Mechanisms of damage include the following: nerve compression from haematoma, pseudoaneurysms or sandbags applied to the injection site; compression and stretching secondary to the use of large-bore delivery sheaths; penetrating damage from direct puncture of the nerve trunk; accumulation of local anaesthetic injected before the procedure around the femoral artery or directly into the nerve myelin sheath.

A few cases of nerve injury secondary to vascular surgery are also reported: one case following crossectomy and stripping of the great saphenous vein [102], one case secondary to percutaneous transluminal angioplasty of the lower limbs [103] and a series of 8 cases following aortofemoral bypass [10].

The pathogenesis of the lesion was identified as a multiple stab avulsion procedure performed on completion of saphenous stripping, nerve compression secondary to bladder distension and nerve compression secondary to haematoma or pseudoaneurysm, respectively.

### 4.6 Iatrogenic femoral nerve injury in anaesthesiological procedures

Regional anaesthesia by femoral nerve block, administered as a single injection or as a continuous infusion through a catheter, is a commonly used procedure to relieve postoperative pain related to hip and knee surgery.

The larger study on the occurrence of femoral nerve injury following single-shot nerve block identified a frequency of 0.03 % (3 cases out of 10,309 procedures) [105].

Iatrogenic femoral nerve injuries following continuous femoral block are reported with a frequency of 0.4 %–0.8 % [110,111,113]

The mechanism of injury is usually the penetration of the needle into the nerve, often in combination with nerve toxicity due to the high doses of anaesthetic introduced.

Iatrogenic lesion of the femoral nerve as a result of ilioinguinal nerve block during inguinal hernia repair occurs frequently. In these circumstances, the anaesthetic agent diffuses from a ventral fascial plane (between the transversus abdominis and the transversalis fascia) to a dorsal plane (between the iliacus and the overlying fascia, where the femoral nerve is located) [116].

### 4.7 Iatrogenic femoral nerve injury in plastic surgery

We found only two reports of iatrogenic femoral nerve injuries occurring in plastic surgery.

One was a medial thigh lift in a formerly obese woman who had lost weight, in which the development of a post-surgical haematoma resulted in a transient femoral neuropathy that resolved within 24 h [120].

The other case was an abdominoplasty complicated by postoperative femoral neuropathy of uncertain origin, probably caused by intraoperative compression, suture ligation, or a side effect of anaesthetic infiltrating the surgical site.

Again, the paralysis resolved very quickly (within 48 h) [121].

### 4.8 Management and prognosis

In the vast majority of cases, a proper course of physical therapy and rehabilitation can restore normal nerve function within a few weeks.

In some cases, such conservative treatment must be preceded by a surgical approach to resolve the anatomical issue underlying the nerve injury (e.g., removal of pseudotumoral masses, evacuation of haematomas, and removal of extruded cement from hip prostheses).

In a small percentage of cases, surgical procedures such as neurolysis and nerve grafting are required.

In general terms, the prognosis for iatrogenic femoral nerve injury is good, with almost complete recovery of nerve function within a few weeks to a few months.

However, ischaemic lesions generally have longer recovery times than compression lesions, due to the concomitant axonal injury [138].

A review of 44 cases quantified the recovery time as one week in 25 % of cases, one month in another 25 % of cases, less than five months in 35 % of cases, and less than one year in the remaining 15 % [139].

In a series of 31 cases of unilateral femoral neuropathy, 31 %, 34 %, and 31 % of excellent, satisfactory, and poor outcomes, respectively, were recorded [140].

The main prognostic factor is, in fact, represented by the estimate of axonal loss, carried out by electroneurophysiology investigations.

Albrecht et al. [50], suggest a possible way to standardise the extent of axonal loss, which involves assessing CMAP (compound muscle action potential) before and after an operation that is particularly at risk of iatrogenic femoral nerve injury. The amount of axonal loss, if any, is calculated using the following formula: 100 × [PreCMAP – PostCMAP] ÷ PreCMAP, establishing 20 % as the axonal deficit cut-off for the definition of femoral neuropathy.

### 4.9 Prevention strategies

One of the most significant features of iatrogenic femoral nerve injury, which is also of intense medico-legal interest, is its preventability, as it can be avoided in most cases by adopting simple preventive measures.

In the case of interventions using self-locking retractors, the appropriate size should be selected and positioned so that they retract only the rectus abdominis without compressing the psoas muscle.

Another useful practice is to periodically check the correct positioning of the valves, given the possibility of small movements of the retractor during surgery.

The shortest blades possible should also be used, especially in a patient with a thin abdominal wall, poorly developed rectus abdominis muscles, and a narrow pelvis [141].

Furthermore, it is important to test the femoral artery pulse after retractor placement, bearing in mind, however, that the finding of a normosfigmic pulse does not rule out the possibility of ischaemic damage.

In the case of surgery on a patient in a lithotomic position, the surgeon must limit flexion, external rotation, and adduction of the hip as much as possible to avoid possible compression of the nerve against the inguinal ligament [64].

With regard to total hip replacement surgery involving the placement of acetabular screws, the screws must be placed in the posterosuperior area of the acetabular cavity to minimise the risk of contact with the vascular-nervous structures [142].

In cases of THA-related nerve injury secondary to compression due to foreign body reactions to wear debris, no specific preventive measures are reported, but close postoperative monitoring is strongly recommended since it can very easily detect by pelvic CT and electromyography any nerve dysfunction and then quickly resolve it before the nerve damage becomes irreversible.

In knee surgery with pneumatic tourniquet, the tourniquet should be applied in the proximal region of the thigh, where the circumference is greater, as this is where the muscle mass is more robust and, therefore, able to offer greater protection to the nerve structure [49].

In major gynaecological surgery, due to the anatomical complexity of the pelvis, a thorough and detailed knowledge of the neuroanatomy of the region is probably the most effective preventive measure.

Apart from this, special attention should be paid to positioning the patient in a lithotomic position, positioning the retractor blades, and avoiding extreme lateral extension of the transverse lower abdominal incisions.

Incorporation of the internal oblique muscle during fascial repair of such wounds should also be avoided.

In the case of nerve blocks, ultrasound-guided technique rather than “blind” blocking is strongly recommended, not only because it allows better topographic localisation of the block, but also because it allows faster absorption of the anaesthetic (requiring a lower dose of drug to be injected) [115].

### 4.10 Medico-legal implications

Iatrogenic lesions of the femoral nerve are of great interest from a medico-legal perspective because of the preventability of the damage, on the one hand, and the particularly favourable characteristics of the dynamics of functional recovery, on the other hand.

Concerning the first aspect, for almost every damage mechanism there are specific preventive measures, which, if properly implemented, can effectively prevent injury.

Compression and traction injuries, which are particularly common in abdominal and orthopaedic surgery, can be easily prevented by careful positioning of retractors and periodic checking of retractor placement, as well as by prudent use of the lithotomy position, which avoids maximal joint angles.

In the case of neurotmesic damages from complete transection, which are very rare and usually associated with pelvic or spinal surgery, the only effective preventive strategy is a thorough anatomical knowledge combined with caution in the surgical act.

A lower coefficient of preventability is associated with compression by haematomas of the iliac and iliopsoas muscles since the occurrence of a muscular haematoma is a subsequent circumstance and not concomitant to surgery. Therefore, the only possibility of effectively combating nerve damage remains scrupulous postoperative monitoring, which must be even more careful in predisposed subjects (e.g., subjects with coagulation disorders or undergoing treatment with anti-platelet/anticoagulants).

Similar considerations apply to ischaemic-type damage, which is essentially compressive damage, from crushing the iliac-femoral vessels against the inguinal ligament. A special case is knee surgery with pneumatic tourniquet, where the compression is directly exerted by the inflation device, and whose potential for neurological damage can be drastically reduced by a prudent choice of filling pressure and dwell time.

From a medico-legal perspective, therefore, the wide margins of preventability of the damage make the iatrogenic lesion of the femoral nerve a complication that can be classified as “preventable and foreseeable” and, therefore, constitutes an essentially objective liability of the healthcare professional and facility, whose only defence should be represented by the demonstration of the intervention of an exceptional and unforeseeable event that played a decisive role in causing the injury.

Regarding the medico-legal assessment of the damage, it is particularly interesting to note that in the vast majority of cases, as can be deduced from the results of this Scoping Review, the damage is reversible, resulting in almost complete functional recovery within 6–12 months, at most, with minor residual deficits.

As our study of the literature demonstrated, the initial damage (close to the surgical insult) is often significantly more severe than the final impairment.

It is, therefore, clear that the medico-legal assessment of permanent damage must be calibrated in relation to a precise temporal dynamic to avoid definitively evaluating impairments that are still evolving.

Electrophysiological investigations are essentially useful in four temporal contexts: intraoperatively (monitoring of action potentials distal to the possible site of injury); in an initial post iatrogenic damage phase, 7–10 days after surgery to distinguish between a simple conduction block and an axonotmesis picture; about one month after surgery in which the damage was determined, when it is possible to characterise the damage more precisely; and 3–4 months after a possible nerve repair operation, while carefully monitoring the evolution of the reinnervation process [143,144].

The timing of detection of abnormalities such as fibrillation potentials and sharp positive waves on EMG is strongly influenced by the length of the nerve tract distal to the site of injury. While such findings are usually detectable about two weeks after injury in the case of short “stumps”, in the case of longer stumps, it can take up to 30 days before electromyographic abnormalities are detectable.

In nerve conduction studies, neuroapraxic lesions are characterised by normal conduction distal to the lesion.

Motor conduction studies on nerves that have undergone axonotmesis and neurotmesis are initially completely superimposable until Wallerian degeneration occurs (usually nine days after injury), which makes it possible to distinguish between the two types of lesions.

Following Wallerian degeneration, there are absent motor responses both proximal and distal to the lesion.

Based on these findings, it is evident that a synergistic use of electromyography and nerve conduction studies allows the precise definition of the degree and probable prognosis of the nerve lesion [145,146].

Determining the time interval that must elapse before a femoral nerve injury can be considered definitively stabilised is a complex matter.

The prognosis of nerve injuries, in general, is strongly influenced by the length of the nerve tract that must undergo regeneration, which is a relatively slow process.

The rate of axonal regrowth of 1 mm per day is only theoretical and can be significantly reduced by several factors, including the formation of scar tissue between stumps.

According to consolidated literature data, the complete regeneration of an injured nerve can take between 3 and 6 years [147].

The possible intervention of atrophy and fibrosis of the denervated muscle fibres must also be considered, which occur within a few weeks and about 1–2 years after the initial injury, respectively.

The absence of nerve potentials 2–3 months after the trauma is considered a negative prognostic sign, indicating the poor possibility of nerve function recovery.

## 5 Conclusion

Iatrogenic femoral nerve injury is a well-known and documented complication of many surgical procedures

A review of the cases reported in the literature over the last 20 years allowed us to precisely define the mechanisms and aetiology of the damage, which in almost all cases can be traced to preoperative or intraoperative conduct characterised by imprudence or inexperience.

We also noted that the lesion is characterised by a decidedly favourable prognosis, with a satisfactory motor and sensory recovery achievable in most cases in 6–12 months and with only rehabilitation treatment, neurolysis, and nerve grafting being reserved for exceptional cases.

This can be explained by the nature of the damage, which in most cases, is neuroapraxic or axonotmesic.

This prognostic behaviour, combined with a complex interpretation of neurophysiological data, makes the medico-legal assessment of impairment sequelae particularly challenging, requiring particular sensitivity and medico-legal expertise.

The results of this research can have a potential impact both on clinical practice, having clearly highlighted how in many cases the injury is preventable and having discussed the methods of prevention, and on the medico-legal methodology, having provided useful elements for the formulation of a more accurate prognostic judgment.

The results of this scoping review may represent the basis for a systematic review.

## Funding statement

The authors received no financial support for the research, authorship, and/or publication of this article.

## Provenance and peer review

Not commissioned, externally peer-reviewed.

## Declaration of competing interest

The authors declare that there is no conflict of interest regarding the publication of this paper.

## Data Availability

The data used to support the findings of this study are included within the article.
